# Constructing a highly bioactive tendon-regenerative scaffold by surface modification of tissue-specific stem cell-derived extracellular matrix

**DOI:** 10.1093/rb/rbac020

**Published:** 2022-04-20

**Authors:** Liang-Ju Ning, Jing Cui, Shu-Kun He, Ruo-Nan Hu, Xuan Yao, Yi Zhang, Wei Ding, Yan-Jing Zhang, Jing-Cong Luo, Ting-Wu Qin

**Affiliations:** 1 Laboratory of Stem Cell and Tissue Engineering, Orthopedic Research Institute, State Key Laboratory of Biotherapy and Cancer Center, West China Hospital, Sichuan University and Collaborative Innovation Center of Biotherapy, Chengdu, Sichuan 610041, P.R. China; 2 Department of Orthopedic Surgery, Orthopedic Research Institute, West China Hospital, Sichuan University, Chengdu, Sichuan 610041, P.R. China; 3 Core Facility, West China Hospital, Sichuan University, Chengdu, Sichuan 610041, P.R. China

**Keywords:** tendon-regenerative scaffold, extracellular matrix, bioactive material, tissue-specific stem cell

## Abstract

Developing highly bioactive scaffold materials to promote stem cell migration, proliferation and tissue-specific differentiation is a crucial requirement in current tissue engineering and regenerative medicine. Our previous work has demonstrated that the decellularized tendon slices (DTSs) are able to promote stem cell proliferation and tenogenic differentiation *in vitro* and show certain pro-regenerative capacity for rotator cuff tendon regeneration *in vivo*. In this study, we present a strategy to further improve the bioactivity of the DTSs for constructing a novel highly bioactive tendon-regenerative scaffold by surface modification of tendon-specific stem cell-derived extracellular matrix (tECM), which is expected to greatly enhance the capacity of scaffold material in regulating stem cell behavior, including migration, proliferation and tenogenic differentiation. We prove that the modification of tECM could change the highly aligned surface topographical cues of the DTSs, retain the surface stiffness of the DTSs and significantly increase the content of multiple ECM components in the tECM-DTSs. As a result, the tECM-DTSs dramatically enhance the migration, proliferation as well as tenogenic differentiation of rat bone marrow-derived stem cells compared with the DTSs. Collectively, this strategy would provide a new way for constructing ECM-based biomaterials with enhanced bioactivity for *in situ* tendon regeneration applications.

**Figure:**
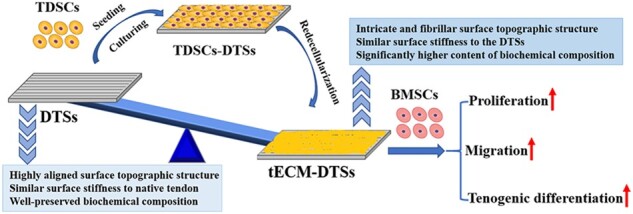

## Introduction

The regeneration of damaged tendons represents a grand challenge in orthopedics because of their limited ability for self-repair. Tissue engineering has become an attractive approach for the treatment of damaged tendons. The classical tissue engineering strategy relies on the use of culture-expanded patient’s own cells and natural and/or synthetic biomaterial scaffolds to produce cell-laden tissue constructs for implantation [1]. However, this approach shows notable limitations, such as the donor-tissue morbidity, the requisite for large number of immune-acceptable cells [2], the long production cycle of engineered tissues *in vitro* as well as the challenges owing to long-term storage and preservation of engineered tissues [3]. Such disadvantages have hindered the clinical application of engineered tendon constructs to repair damaged tendons by the classical tissue engineering strategy.

Latest advances in tissue engineering and regenerative medicine have employed a new strategy to harness the potential of endogenous stem/progenitor cells for *in situ* tissue repair and regeneration [1, 4, 5]. Much attention has been focused on the design of biomaterials for *in situ* tissue regeneration to recruit endogenous stem cells to the injury site. Several studies have proved that the incorporation of stromal cell-derived factor-1 (SDF-1) into scaffold materials via factor adsorption, mini-osmotic pump delivery or genetic engineering method of collagen-binding domain could enhance the recruitment of endogenous stem cells to the injury site [6, 7]. In another study, Kim and colleagues demonstrated modifying self-assembling peptide nanofiber using substance P sequence was able to recruit endogenous mesenchymal stem cells (MSCs) [8]. Nair and colleagues found that the biomaterials with varying degrees of pro-inflammatory properties triggered different extents of endogenous stem cell recruitment, and these recruited cells arriving at the implant sites were multipotent [9]. This reminds us that the scaffold material with the capacity to recruitment of stem cells alone was not enough. The success of *in situ* tissue regeneration not only depends on efficient recruitment of host stem/progenitor cells into the implanted scaffold materials but also needs to effectually induce the recruited stem cells into tissue-specific cell lineages [1]. Lu *et al*. [10] reported that the oriented acellular cartilage matrix scaffold modified by bone marrow homing peptide could increase the recruitment of endogenous stem cells and chondrogenic differentiation, resulting in a significant improvement in the repair of chondral defects. These previous findings highlighted the necessity of recruiting abundant endogenous stem cells and inducing them to differentiate into tissue-specific cell lineages for tissue regeneration.

Recently, cell-derived extracellular matrix (ECM), especially stem cell-derived ECM, attracted increasing attention in the area of tissue engineering and regenerative medicine [11–14]. Decellularized ECM from *in vitro* stem cell cultures has been proved to provide an instructive stem cell microenvironment that can rejuvenate aged progenitor cells, promote stem cell expansion and direct stem cell differentiation [13, 15, 16]. Either on its own or integrated with other scaffold materials, stem cell-derived ECM also can be used as biomaterials to produce tissues *de novo* or promote endogenous regeneration [17–19]. To date, multiple stem cell-derived ECM, including pluripotent stem cells [20, 21], bone marrow-derived stem cells (BMSCs) [16, 18, 22], synovium-derived stem cells (SDSCs) [13, 15], adipose tissue-derived stem cells [23, 24], dental pulp stem cells (DPSCs) [25], umbilical cord MSCs [26] and so forth, have been extensively studied over the past decades. Our recent studies demonstrated that the scaffolds modified with ECM of tendon-derived stem cells (TDSCs) markedly improved BMSCs migration *in vitro* and could recruit more endogenous stromal cells for accelerating healing of the tendon-bone interface *in vivo* [27, 28]. Nevertheless, the information on the tendon-specific stem cell-derived ECM (tECM) is still scarce and no studies have systematically investigated the use of tECM for constructing a highly bioactive tendon-regenerative scaffold.

In our previous studies, we have proved that the decellularized tendon slices (DTSs) that retained the native tendon ECM microenvironment cues are able to promote stem cell proliferation and tenogenic differentiation *in vitro* and show certain pro-regenerative capacity for rotator cuff tendon regeneration *in vivo* [29–31]. In the present study, we present a strategy to further improve the bioactivity of the DTSs for constructing a novel highly bioactive tendon-regenerative scaffold by surface modification of the tECM (i.e. tECM-DTSs), which is expected to greatly enhance the capacity of scaffold material in regulating stem cell behavior, including migration, proliferation and tenogenic differentiation. In detail, the surface topography, and surface nanomechanical properties and biochemical components of the tECM-DTSs were first characterized, and then the regulatory capacity of the tECM-DTSs to the migration, proliferation and tenogenic differentiation of rat BMSCs was investigated. It was hypothesized that tECM could confer higher bioactivity to the DTSs so as to endow the tECM-DTSs with a greater capacity to enhance the migration, proliferation as well as tenogenic differentiation of rat BMSCs.

## Materials and methods

### Cell isolation and culture

We used male Sprague Dawley rats (4–5 weeks old, 100–120 g weight) for the isolation and culture of TDSCs and BMSCs with approval from the Animal Care and Use Committee of Sichuan University. The procedures for the isolation and culture of TDSCs and BMSCs were same as our previously published protocols [30].

### Fabrication of the tECM-DTSs

**Figure 1.**
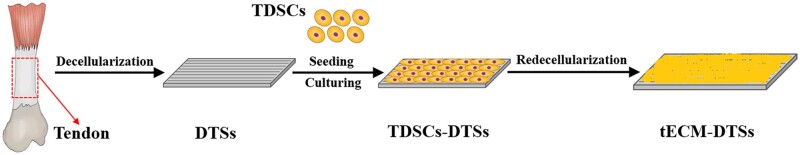
Schematic illustration of the fabrication of the tECM-DTSs. The Achilles tendons of adult beagle dogs were decellularized to prepare the DTSs substrate, and TDSCs were seeded on the top surface of DTSs substrate to construct the composites of TDSCs-DTSs, and then these composites were re-decellularized to fabricate the tECM-DTSs

A typical process for the fabrication of the tECM-DTSs is presented in Fig. 1. First, the DTSs substrate was fabricated using our previously published protocol [29]. In short, the Achilles tendons of adult beagle dogs were decellularized through the following procedures: repetitive freeze/thaw treatment, frozen section with a thickness of 300 μm and nuclease treatment (including DNase 150 IU/ml and RNase 100 μg/ml) for 12 h at 37°C. Following washing in 50 ml of 0.1 M PBS (3 × 30 min), the DTSs were lyophilized and sterilized with ethylene oxide (EO). Then, TDSCs were seeded on the top surface of DTSs substrate at 1 × 10^5^ cells per cm^2^ and cultured in complete medium supplemented with 20% fetal bovine serum (FBS). After reaching 90% confluence, 50 μM of L-ascorbic acid phosphate (Sigma) was added for additional culture period of 8 days. At the end of 15-day culture period, the composites of TDSCs-DTSs were re-decellularized as described previously with minor alteration [15], using 0.5% Triton X-100 supplemented with 20 mM ammonium hydroxide (NH_4_OH) at 37°C for 15 min, followed by 100 U/ml DNase I at 37°C for 2 h. Finally, the tECM modified DTSs (hereafter referred to as tECM-DTSs) were washed in 50 ml of 0.1 M PBS (6 × 30 min), frozen at –80°C or lyophilized and sterilized by EO for subsequent use.

### Evaluation of redecellularization

For DNA quantification, lysates of the lyophilized samples (*n* = 10 for each group) were prepared by digestion in Proteinase K solution (1 mg/ml, Sigma) at 50°C for 24 h. Residual DNA in the lysates was extracted using our previously published protocol [32], and then measured using the PicoGreen assay according to the manufacture instructions (Invitrogen).

For histological analysis, the frozen samples (*n* = 4 for each group) were fixed, embedded, and stained with hematoxylin and eosin (H&E), Masson or 4,6-diamidino-2-phenylindole (DAPI).

### Scanning electron microscopy

For surface topography characterization, the frozen samples (*n* = 3 for each group) were fixed, sputter coated with gold and examined under scanning electron microscopy (SEM) (FEI Inspect F50) at an accelerating voltage of 30 kV.

### Atomic force microscopy assay

To characterize the nanomechanical properties of the microenvironment provided by the DTSs and tECM-DTSs respectively, the surface stiffness of these specimens (*n* = 5 for each group) was measured using atomic force microscopy (AFM) as our previously published protocol [30].

### ELISA measurements

Cytokines retained in the DTSs and tECM-DTSs, including transforming growth factor beta 1 (TGF-β1), vascular endothelial growth factor (VEGF), insulin-like growth factor 1 (IGF-1) and SDF-1, were measured using ELISA. Soluble molecules were extracted from the DTSs and tECM-DTSs specimens using Tissue Extraction Reagent I (FNN0071, Thermo Fisher Scientific, USA) with a protease inhibitor (1% phenylmethane sulfonyl fluoride, 1% PMSF, Sigma) at 4°C for 24 h. The extracted lysates were homogenized, and centrifuged at 10 000 rpm for 10 min at 4°C, and then the supernatants were collected. ELISA measurements of the extracted lysates were performed (*n* = 6 for each group) according to the manufacturer’s instructions (TGF-β1 and VEGF, NeoBioscience, China; IGF-1, RayBiotech, USA; SDF-1, DL-develop, China).

### Western blot analysis

For western blot analysis of critical tendon ECM components in the DTSs and tECM-DTSs, the lyophilized samples (*n* = 3 for each group) were minced and homogenized using the RIPA Lysis Buffer (Beyotime, China) supplemented with 1% PMSF. Total proteins were quantified using the BCA Protein Quantification kit (Beyotime Biotechnology, China). Thirty micrograms of protein from each sample was loaded onto SDS-PAGE gel for electrophoresis, and then transferred to 0.2 μm polyvinylidene fluoride (PVDF) membranes (Millipore) by wet electroblotting. The membrane was incubated with the following primary antibodies: rabbit anti-biglycan (1:1000, Abcam), rabbit anti-fibromodulin (1:1000, GeneTex), mouse anti-fibronectin (1:1000, Abcam), rabbit anti-vitronectin (1:1000, Abcam) or rabbit anti-glyceraldehyde-3-phosphate dehydrogenase (GAPDH, 1:1000, Abcam) at 4°C overnight. Then, the membranes were washed in TBST buffer for three times and incubated with corresponding secondary antibodies of horseradish peroxidase (HRP) conjugated goat anti-rabbit or goat anti-mouse IgG (Western Biotechnology, China) for 1.5 h at room temperature. Finally, these membranes were incubated with chemiluminescence substrate (Shanghai ShineGene Molecular Biotech., China), and exposed to two stacked blue x-ray films (Kodak) in a cassette. After scanning the film, semi-quantification of band intensity was performed with UVP gel image processing system Labworks 4.6 software, and the relative protein expression level was normalized to the band intensity of GAPDH.

For western blot analysis of differentiation-related proteins expression in BMSCs induced by the DTSs and tECM-DTSs, the expression of tendon-specific markers on the protein level was examined in BMSCs cultured on the DTSs and tECM-DTSs in complete culture media (10% FBS) for 3, 7 and 14 days. At the designated time points, total proteins (*n* = 3 for each group) were extracted and quantified. After protein transfer, the PVDF membranes were incubated using the following primary antibodies: rabbit anti-scleraxis (SCX, 1:1000, Bioss), rabbit anti-tenomodulin (TNMD, 1:1000, Abcam), rabbit anti-thrombospondin-4 (THBS4, 1:1000, Abcam) or mouse anti-β-actin (1:2000, Servicebio), followed by incubation with the HRP-conjugated secondary antibodies (Servicebio, China). Then, these membranes were incubated with enhanced chemiluminescence solutions (ECL, Servicebio, China) and the target protein bands were imaged with a chemiluminescence imaging system (ChemiScope 6300, Clinx, China). Semi-quantification of band intensity was performed with AlphaEaseFC software (Alpha Innotech, USA), and the relative protein expression level was normalized to the band intensity of β-actin.

### Cell migration assay

For cell migration assay, the conditioned medium of the DTSs and tECM-DTSs was prepared as previously described with some alteration [33]. Briefly, the DTSs or tECM-DTSs samples were incubated in 1% W/V of DMEM containing 5% FBS for 72 h at 37°C to make the conditioned medium for each material. Transwell migration chambers (Corning, USA) with 8 μm pore size were used to evaluate the migration ability of BMSCs regulated by the DTSs and tECM-DTSs. After serum-starvation overnight, BMSCs were harvested and counted, and 1 × 10^4^ cells were resuspended in 200 μl of medium with 5% FBS and added into the upper chambers. To induce chemotaxis, 1 ml of the conditioned medium from the DTSs or tECM-DTSs was added to the lower chambers. After incubation at 37°C for 48 h, the cells that migrated to the lower side of the membrane were fixed in 4% paraformaldehyde, stained with DAPI and quantified with ImageJ software (NIH). Five randomly selected fields of each sample (*n* = 4 for each group) were counted at 200× magnification under an inverted fluorescence microscope (Nikon, Japan).

### Cell proliferation assay

To investigate the effect of soluble factors released from the DTSs and tECM-DTSs on cell proliferation, the conditioned medium was prepared as described above. BMSCs were seeded in wells of 96-well plates at a density of 5 × 10^3^ cells per well. After the cells had attached, the medium was replaced with the conditioned medium from the DTSs or tECM-DTSs. The wells with non-conditioned medium only served as blank control. After 1, 2 and 3 days of incubation, cell viability (*n* = 4) was measured using the alamarBlue assay following the manufacturer’s protocol (Invitrogen).

To further investigate the effect of DTSs and tECM-DTSs themselves on cell proliferation, BMSCs were directly seeded on the DTSs and tECM-DTSs at 2 × 10^5^ cells per cm^2^ and incubated for a period of 3 days. The cell viability was qualitatively assessed using LIVE/DEAD cell staining assay as described previously [30]. Images of live and dead cells were acquired under an inverted fluorescence microscope (Nikon, Japan). Subsequently the cell morphology and alignment from these samples were observed using SEM.

### Real-time quantitative reverse transcription PCR

For real-time quantitative reverse transcription PCR (RT-qPCR) analysis, total cellular RNA (*n* = 6 for each group) was extracted at the designated time points (3, 7 or 14 days) using TRIzol (Invitrogen, Carlsbad, CA). Reverse transcription was achieved using the First Strand cDNA kit according to the manufacturer’s protocol (Promega, Madison, WI, USA). qPCR was performed using the SYBR Green PCR master mix (TakaRa, Japan) with specific primers on a Light Cycler system (Roche, Switzerland). Rat-specific primers for tendon-specific genes, including *SCX*, *TNMD*, *THBS4*, and tendon-related genes, including *TNC*, *COL I* and *COL III*, and the housekeeping gene, *GAPDH*, were synthesized by Sango Biotech (Shanghai, China). The primer sequences for the tested genes are listed in Table 1. The cycling conditions were as follows: denaturation at 95°C for 2 min, 45 cycles at 95°C for 10 s, optimal annealing temperature (shown in Table 1) for 10 s and 72°C for 10 s. The relative expression level of each target gene was determined using the 2^–ΔΔCt^ method.

**Table 1.** Primer sequences, product size and annealing temperature used for PCR analysis

Genes	5′-3′ Primer sequences	Production size (bp)	Annealing temperature (°C)
*GAPDH*	Forward GCAAGTTCAACGGCACAG	140	60
	Reverse GCCAGTAGACTCCACGACAT		
*SCX*	Forward AGAACACCCAGCCCAAACA	111	59
	Reverse GTGGACCCTCCTCCTTCTAAC		
*TNMD*	Forward GGACTTTGAGGAGGATGG	128	57
	Reverse CGCTTGCTTGTCTGGTGC		
*THBS4*	Forward AATACCATCCCTGCTACCC	163	60
	Reverse TTCCGACACTCGTCAACA		
*TNC*	Forward AACCACAAGAAATAACCCTC	137	59
	Reverse TGTTGCTATGGCACTGACT		
*COL I*	Forward CGAGTATGGAAGCGAAGG	101	58
	Reverse AGTGATAGGTGATGTTCTGG		
*COL III*	Forward CTCCCAGAACATTACATACCA	189	58
	Reverse GTCTTGCTCCATTCACCAG		

*COL I*, *collagen Type I*; *COL III*, *collagen Type III*; *GAPDH*, *glyceraldehyde-3-phosphate dehydrogenase*; *SCX*, *scleraxis*; *THBS4*, *thrombospondin-4*; *TNC*, *tenascin-C*; *TNMD*, *tenomodulin*.

### Statistical analysis

All data were statistically analyzed using SPSS 16.0 software and presented as mean ±SD. For multiple-group comparisons, the data were analyzed using one-way analysis of variance followed by Dunnett’s T3 *post hoc* test. For two-group comparisons, the data were analyzed using the unpaired Student’s t-test. A value of *P *<* *0.05 was considered statistically significant.

## Results

### Confirmation of redecellularization effectiveness

**Figure 2.**
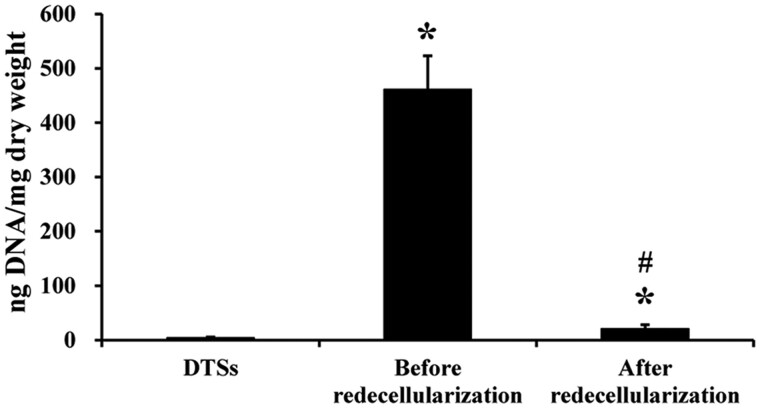
Assessment of redecellularization effectiveness by DNA quantification. PicoGreen analysis of DNA content in DTSs, before and after redecellularization of the composites of TDSCs and DTSs substrate. *P <* *0.05 as compared with the DTSs; ^#^*P *<* *0.05 as compared with before redecellularization

**Figure 3.**
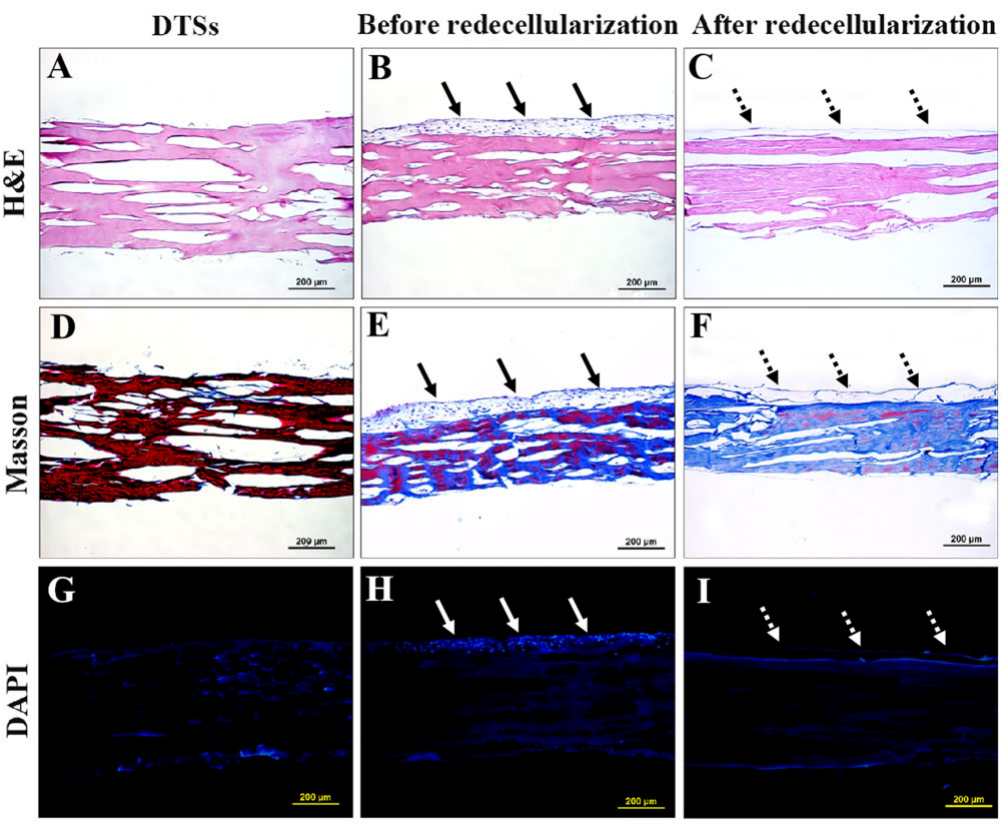
Assessment of redecellularization effectiveness by histological staining. Representative H&E (**A**–**C**), Masson (**D**–**F**) and DAPI (**G**–**I**) stained sections of the DTSs, before and after redecellularization of the composite of TDSCs and DTSs substrate. The solid arrows show the dense cell sheets formed by TDSCs on the top surface of the DTSs at the end of 15 days of culture before redecellularization. The dotted arrows show the tECM deposited on the DTSs substrate after redecellularization. Scale bar = 200 μm

The protocol for redecellularization of the composites of TDSCs and DTSs substrate was effective in removal of the cellular and nuclear components. The PicoGreen assay indicated the residual DNA content was significantly decreased after redecellularization (Fig. 2). Before redecellularization, the composites of TDSCs and DTSs substrate had 460.83 ± 62.15 ng/mg of DNA, which was decreased to 20.60 ± 7.84 ng/mg after redecellularization (Fig. 2). As shown in Fig. 3, histological analysis further confirmed that TDSCs formed dense cell sheets on the top surface of the DTSs at the end of 15 days of culture before redecellularization (Fig. 3B, E and H), whereas the cellular and nuclear material were efficiently removed and tECM was effectively deposited on the DTSs substrate after redecellularization (Fig. 3C, F and I).

### Surface topography, stiffness and biochemical components of the tECM-DTSs

**Figure 4.**
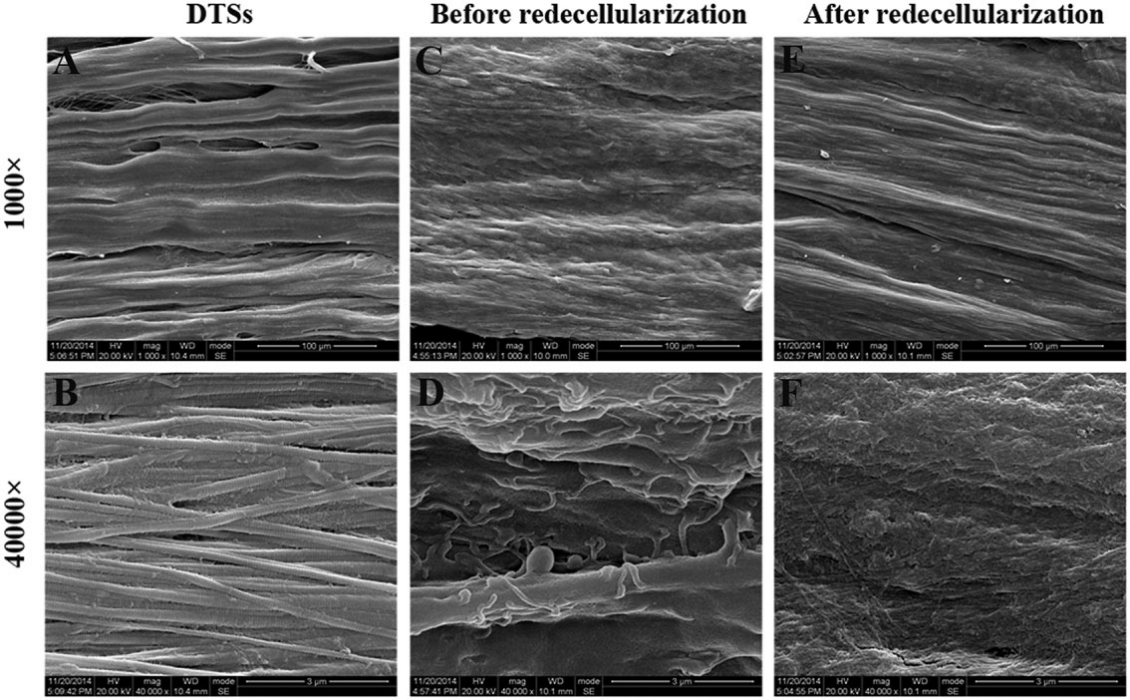
Characterization of surface topography of the DTSs and tECM-DTSs. Representative SEM images of the DTSs (**A** and **B**), before (**C** and **D**) and after redecellularization (i.e. the tECM-DTSs, **E** and **F**) of the composites of TDSCs and DTSs substrate. Scale bar = 100 μm in images A, C and E; scale bar = 3μm in images B, D and F

**Figure 5.**
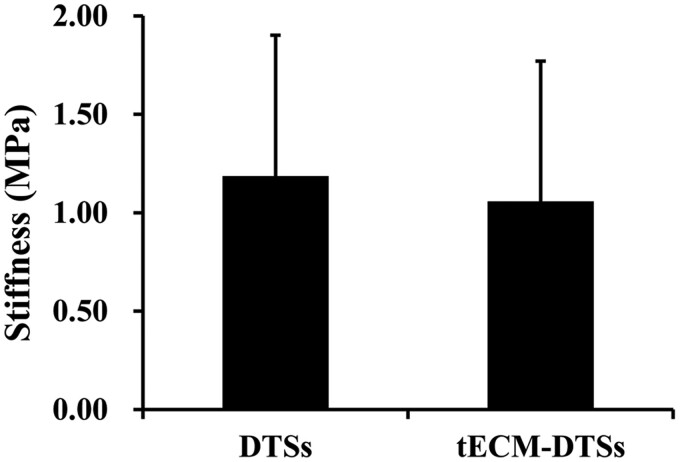
Characterization of surface stiffness of the DTSs and tECM-DTSs. The surface stiffness of the indicated specimens was measured by AFM (*P *>* *0.05)

**Figure 6.**
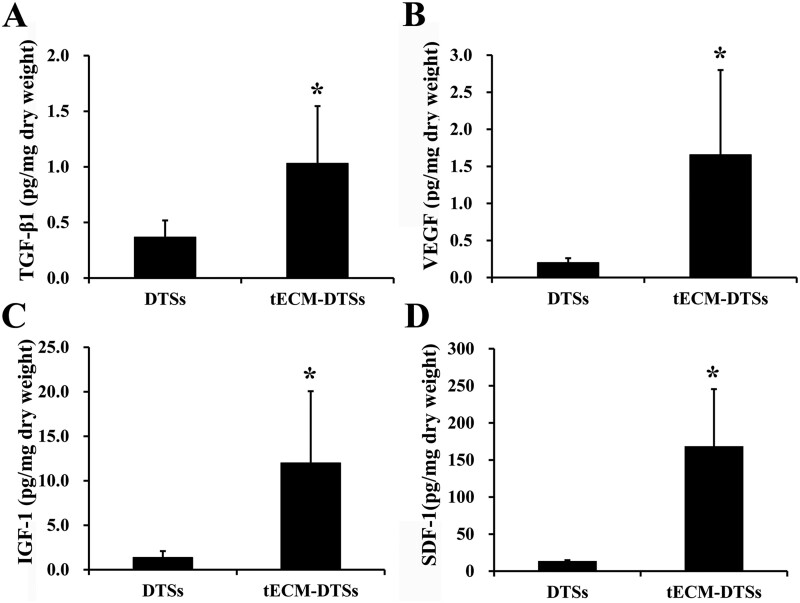
Analysis of bioactive factors retained in the DTSs and tECM-DTSs by ELISA measurements. (**A**) TGF-β1, (**B**) VEGF, (**C**) IGF-1 and (**D**) SDF-1. *P <* *0.05 as compared with the DTSs

**Figure 7.**
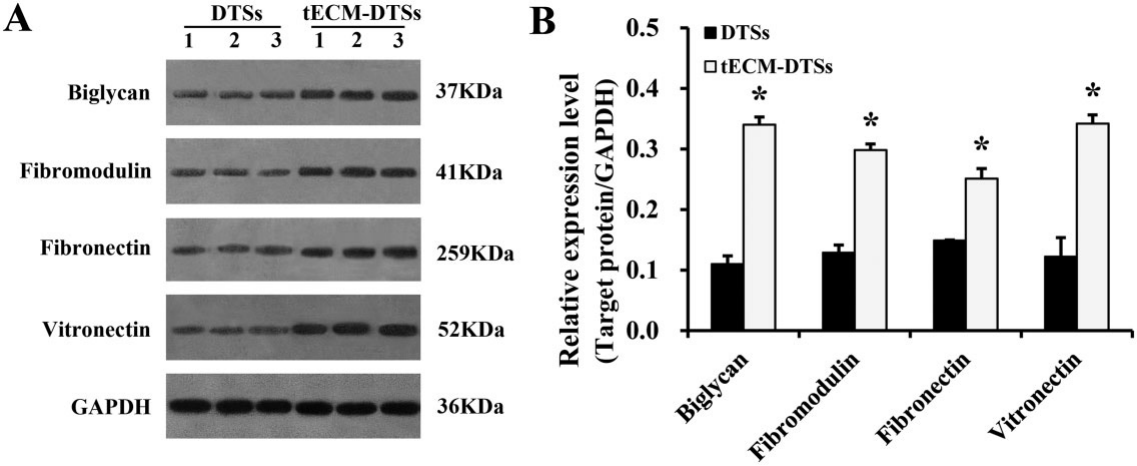
Analysis of critical tendon ECM components in the DTSs and tECM-DTSs by Western blot analysis. (**A**) Western blot images of biglycan, fibromodulin, fibronectin and vitronectin in the DTSs and tECM-DTSs. (**B**) Semi-quantitative analysis of relative expression level of four target proteins in the DTSs and tECM-DTSs. *, *P *<* *0.05 as compared with the DTSs

SEM observation showed obvious changes of the surface topography before and after modification with tECM (Fig. 4). Before modification, the surfaces of the DTSs were well aligned collagen fibers (Fig. 4A) and revealed the typical banding pattern under high magnification (Fig. 4B). When cultured with TDSCs for 15 days, the top surfaces of the DTSs were entirely covered by the dense cell sheets formed by TDSCs before redecellularization (Fig. 4C and D), which are more evident in higher magnification SEM micrographs (Fig. 4B and D). After redecellularization, there was a large amount of tECM deposited on the top surface of the DTSs so that the tECM-DTSs changed the highly aligned surface topographical cues of the DTSs and displayed an intricate and fibrillar ultrastructure (Fig. 4E and F). The results of AFM assay indicated that the surface stiffness of the tECM-DTSs was 1.06 ± 0.71 MPa, which was close to that of the DTSs at 1.19 ± 0.72 MPa (*P *>* *0.05, Fig. 5). ELISA measurements revealed that the levels of multiple cytokines, including TGF-β1 (Fig. 6A), VEGF (Fig. 6B), IGF-1 (Fig. 6C) and SDF-1 (Fig. 6D) in the tECM-DTSs were significantly higher than those in the DTSs (*P *<* *0.05). Compared to that of DTSs, the content of TGF-β1 in the tECM-DTSs increased by 1.81-fold, VEGF by 7.34-fold, IGF-1 by 7.78-fold and SDF-1 by 11.23-fold. Western blot analysis indicated that four critical tendon ECM components (including biglycan, fibromodulin, fibronectin and vitronectin) in the tECM-DTSs were significantly higher than those in the DTSs (*P *<* *0.05, Fig. 7A and B).

### Enhanced cell migration induced by the tECM-DTSs

**Figure 8.**
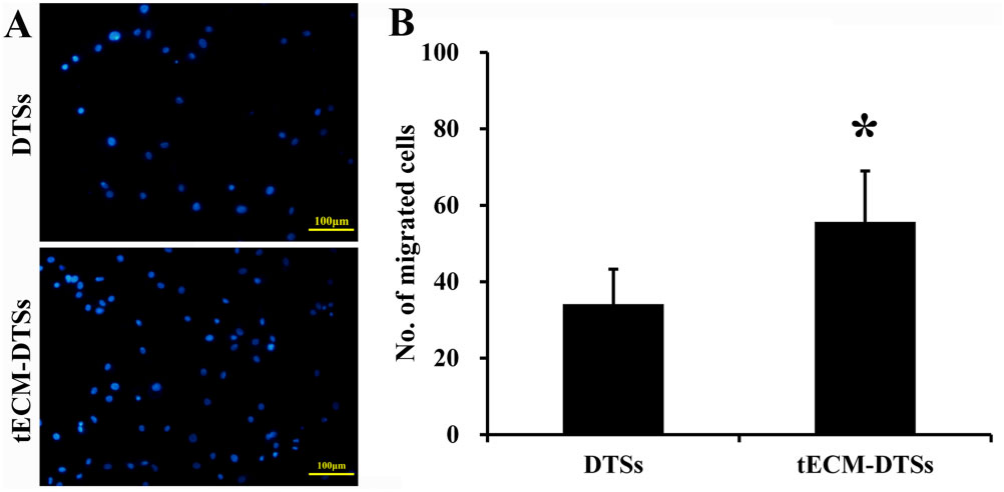
Cell migration assays of the DTSs and tECM-DTSs by Transwell migration assay. (**A**) DAPI staining of the migrated BMSCs for the two groups. Scale bar = 100μm. (**B**) The quantitative analyses of the number of migrated BMSCs for the two groups. *P <* *0.05 as compared with the DTSs

Enhanced bioactivity was first evidenced by the enhanced BMSCs migratory responses to factors released from the tECM-DTSs. As shown in Fig. 8A and B, DAPI staining of the migrated BMSCs for the two groups and quantitative analyses revealed that the number of BMSCs that migrated toward the conditioned medium of the tECM-DTSs was significantly more than toward the conditioned medium of the DTSs (*P *<* *0.05).

### Enhanced cell proliferation induced by the tECM-DTSs

**Figure 9.**
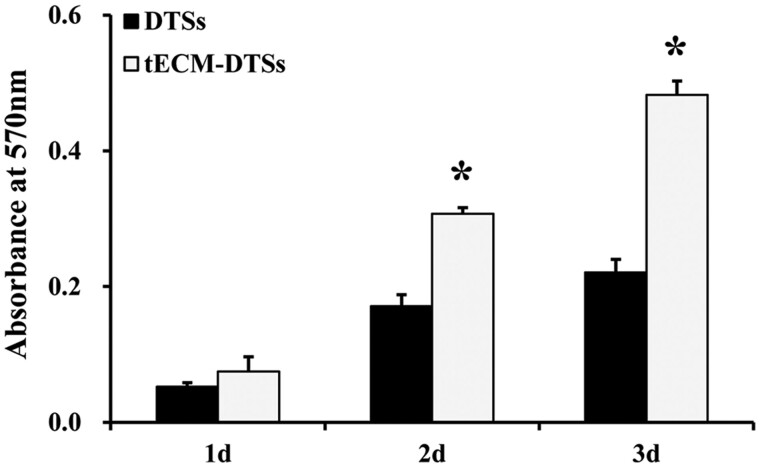
Cell proliferation assays of the DTSs and tECM-DTSs. AlamarBlue assay of cell proliferation of BMSCs cultured in the conditioned medium from the DTSs and tECM-DTSs at 1, 2 and 3 days. *P <* *0.05 as compared with the DTSs

**Figure 10.**
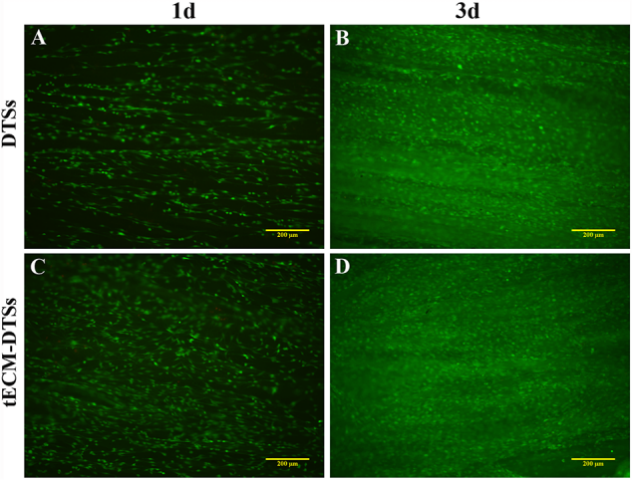
Cell proliferation assays of the DTSs and tECM-DTSs. Live/dead staining of BMSCs directly seeded on the DTSs (**A** and **B**) and tECM-DTSs (**C** and **D**) at 1 and 3 days using fluorescence microscopy. Scale bar = 200 μm

**Figure 11.**
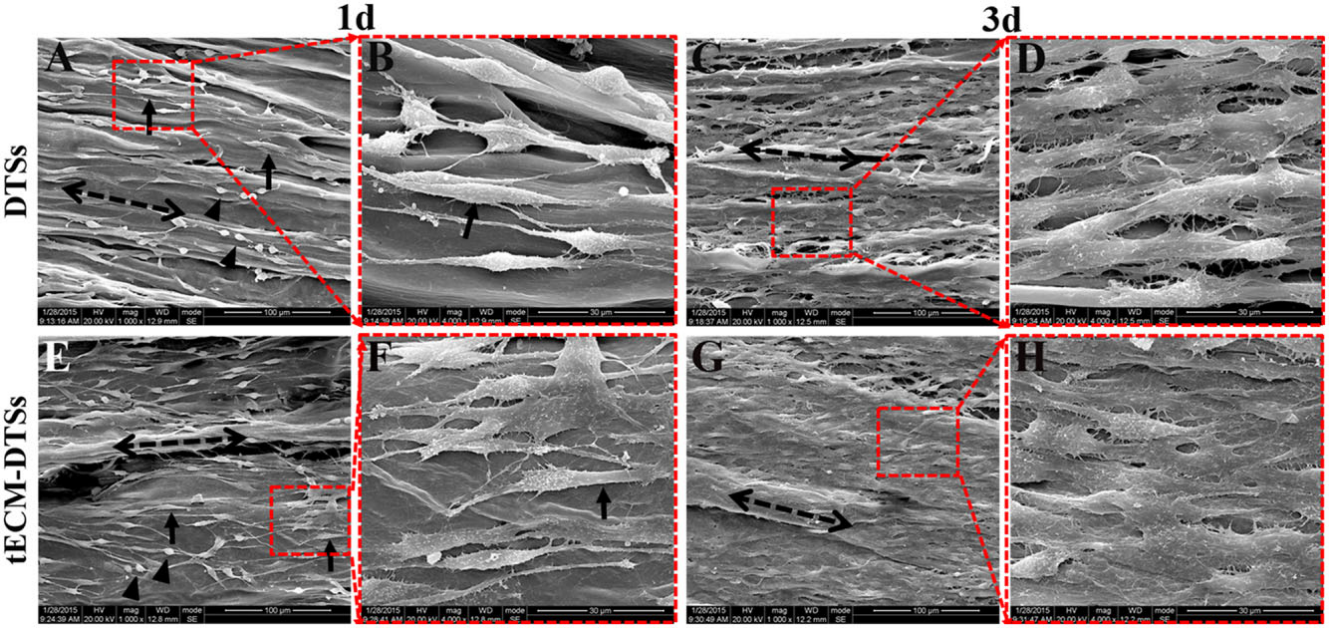
Cell morphology and alignment assays on the DTSs and tECM-DTSs. SEM images of morphology and alignment of BMSCs on the surface of the DTSs (**A–D**) and tECM-DTSs (**E**–**H**) after 1 and 3 days of culture. The dotted arrows represent the direction of collagen fibrils of the DTSs substrate. The solid arrows show the elongated spindle morphology BMSCs. The arrowheads show the polygonal morphology BMSCs. Scale bar = 100 μm for low-magnification images (A, C, E and G); scale bar = 30 μm for high-magnification images (B, D, F and H)

AlamarBlue assay revealed that there was higher but not statistically significant cell viability in tECM-DTSs group when compared with the DTSs group on the Day 1. On the Days 2 and 3, the conditioned medium from the tECM-DTSs significantly promoted the proliferation of BMSCs as compared with that from the DTSs (Fig. 9). When BMSCs were seeded directly on the surface of the tECM-DTSs or DTSs at a moderate cell density, these cells grew robustly on these materials from 1 to 3 days and showed excellent viability, as indicated by the results of live/dead staining (Fig. 10A–D). The SEM images showed that BMSCs were firmly attached to the surface of the DTSs and tECM-DTSs and displayed elongated spindle morphology or spherical morphology after 1 day of culture (Fig. 11A, B, E and F). Specially, the cells on the DTSs were aligned along the collagen fibrils, whereas the cells on the tECM-DTSs showed random orientation. By 3 days, the cells formed dense confluent cell layers on the surface of the DTSs and tECM-DTSs (Fig. 11C, D, G and H), indicating distinct cell proliferation with time extending. Notably, the cell layers on the tECM-DTSs seemed to be denser than those on the DTSs, which was more prominent in higher magnification images (Fig. 11D and H). Overall, these results revealed that the surfaces of the tECM-DTSs are more conducive to BMSCs growth and proliferation, compared to the DTSs.

### Enhanced tenogenic differentiation induced by the tECM-DTSs

**Figure 12.**
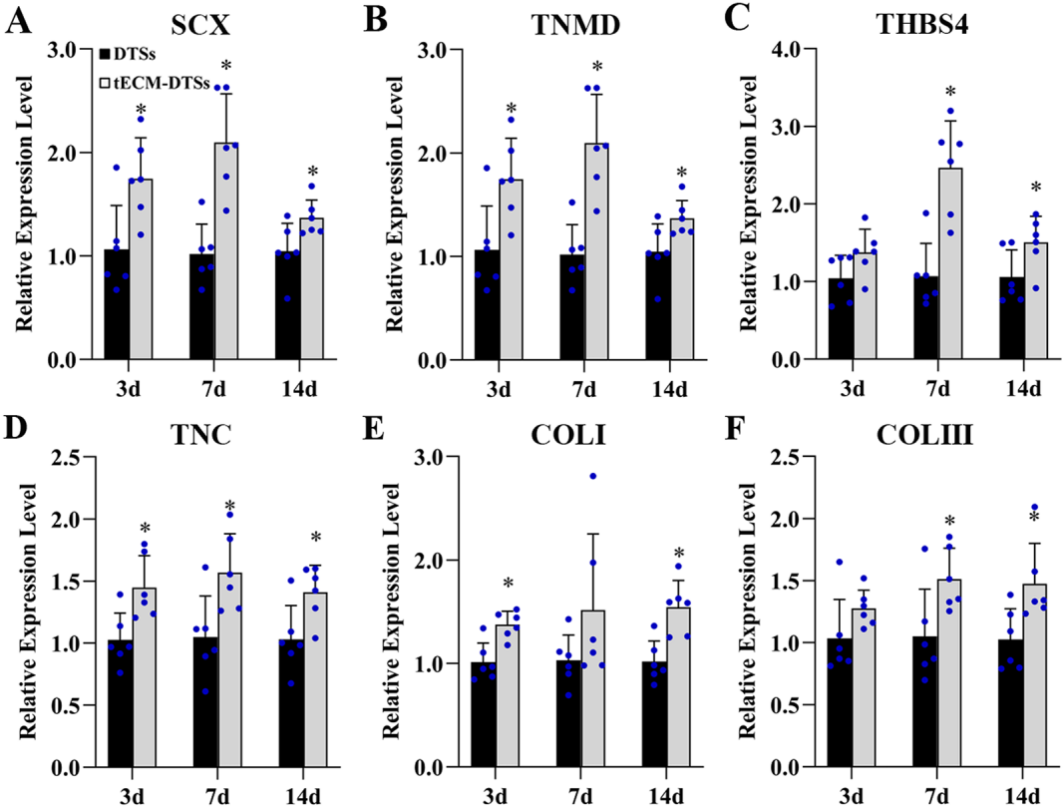
Cell differentiation assays of the DTSs and tECM-DTSs on the gene expression level. (**A**–**F**) RT-qPCR analysis of tenogenic differentiation of BMSCs cultured on the DTSs and tECM-DTSs at different time points. Data are normalized to *GAPDH*. *P <* *0.05 as compared with the DTSs

Tenogenic differentiation of BMSCs cultured on the DTSs and tECM-DTSs at the 3 day, 7 day and 14 day time-points was studied using the RT-qPCR and western blot analysis. On the gene expression level, the expressions of *SCX*, *TNMD* and *TNC* were significantly up-regulated in BMSCs cultured on the tECM-DTSs compared to those on the DTSs at all three time points (Fig. 12A, B and D). Although there was no significant difference between two groups at 3 days, the expressions of *THBS4* and *COL III* were elevated significantly in BMSCs cultured on the tECM-DTSs at 7 or 14 days (Fig. 12C and F). The expression of *COL I* was significantly enhanced in BMSCs cultured on the tECM-DTSs at 3 or 14 days when compared to those on the DTSs, and no significant difference was found at 7 days (Fig. 12E). On the protein expression level, the expression of SCX exhibited relatively higher levels in the tECM-DTSs group than in the DTSs group at all three time points, though no significant difference was found between two groups (supplementary Fig. S1A and B). TNMD expression was significantly higher in the tECM-DTSs group than in the DTSs group at 3 and 7 days, but the difference between the two groups was negligible at 14 days (supplementary Fig. S1A and B). Unexpectedly, the BMSCs cultured on the DTSs and tECM-DTSs showed detectable but low expression levels of THBS4 at all three time points, and no significant difference was observed between the two groups (supplementary Fig. S1A and B). As a whole, these data suggested that the tECM-DTSs displayed greater ability in promoting tenogenic differentiation of stem cells than the DTSs.

## Discussion

The goal of the current study was to develop a novel highly bioactive tendon-regenerative scaffold (i.e. tECM-DTSs) by surface modification of tissue-specific stem cell-derived ECM on the DTSs, which is expected to have a greater capacity in regulating stem cell behavior with the ultimate purpose of recruiting abundant endogenous stem cells and inducing them toward tenogenic differentiation to promote *in situ* tendon regeneration. The results presented here demonstrated that the tECM-DTSs, with similar surface stiffness and higher content of multiple ECM components, showed higher bioactivity in inducing the migration, proliferation and tenogenic differentiation of rat BMSCs, compared to the DTSs.

TDSCs, as tendon tissue-specific stem cells, showed more advantages than other MSCs for musculoskeletal tissue regeneration [34, 35]. Hence, in the current study, TDSCs were chosen to develop the stem cell-derived ECM modified scaffold. TDSCs were seeded on the top surface of the DTSs substrate to form a dense cell sheet and then the composites of TDSCs-DTSs were re-decellularized to develop the tECM-DTSs. It is worth noting that ascorbic acid-2-phosphate is essential supplement for robust ECM deposition [13, 16], after TDSCs were close to 100% confluence on the surface of DTSs. The results of the PicoGreen assay indicated the average DNA content before redecellularization was significantly increased to 460.83 ± 62.15 ng/mg compared to 3.90 ± 1.70 ng/mg of the DTSs, suggesting that TDSCs were successfully seeded on the DTSs substrate and grew well. After redecellularization, the average DNA content of the tECM-DTSs was decreased to 20.60 ± 7.84 ng/mg, though significantly higher than that of DTSs (3.90 ± 1.70 ng/mg). It is currently well accepted that the amount of DNA <50 ng per mg dry weight is the acceptable range for decellularized ECM scaffold material [36]. Our redecellularization protocol was modified from one published protocol that had been widely used in preparation of SDSC- or BMSC-derived ECM [13, 37]. In the pre-experiment phase, the published protocol (Step 1: 0.5% Triton X-100 containing 20 mM NH_4_OH at 37°C for 5 min; Step 2: 100 U/ml DNase at 37°C for 1 h) was attempted to use for redecellularization of the composites of TDSCs-DTSs. Unexpectedly, this protocol did not markedly decrease the DNA content after redecellularization (data not shown). Therefore, we modified this protocol by extending the treatment period of Triton X-100/NH_4_OH as well as DNase, and confirmed the efficiency of the modified protocol. In addition to the PicoGreen assay, the results of histological staining, including H&E, Masson and DAPI staining, also confirmed the modified protocol could effectively remove the cellular components, and also proved that visible tECM was present on the DTSs surface. The results of SEM analysis further verified that a large amount of tECM was indeed deposited on the surface of the DTSs after redecellularization. Notably, the tECM-DTSs displayed different surface topography and ceased to be the well aligned collagen fibrils and the typical banding pattern of the DTSs. AFM assay showed that the surface stiffness of the tECM-DTSs was close to that of the DTSs, namely that the tECM-DTSs also had similar stiffness to native tendon [36]. The results of ELISA and western blot assays showed that four important cytokines (including TGF-β1, VEGF, IGF-1 and SDF-1) and four crucial ECM proteins (including biglycan, fibromodulin, fibronectin and vitronectin) were present in the tECM-DTSs and the content of all these ECM components was significantly higher than that in the DTSs. Though TGF-β1 has been reported to have no direct effect on BMSCs recruitment in a previous study of Zhang *et al*. [38], several other studies demonstrated that the expression of TGF-β1 was increased at the site of tissue injury, which facilitated the homing of BMSCs *in vivo* [39–41]. Dubon *et al*. [42] found that TGF-β1 induced BMSCs migration through *N*-cadherin and noncanonical TGF-β signals. In addition, TGF-β1 also can promote the proliferation of BMSCs via activation of Wnt/β-catenin pathway and/or FAK-Akt-mTOR pathway [43, 44]. VEGF was proved to regulate BMSC migration and proliferation through stimulating platelet-derived growth factor receptors [45]. IGF-1 was found to promote stem cell recruitment via paracrine release of SDF-1 [46], and SDF-1 has been widely demonstrated to regulate stem cell homing, which plays a crucial role in tissue repair and regeneration [6, 47, 48]. Biglycan and fibromodulin, as two critical components that organize the TDSCs niche, their absence could detour TDSCs fate from tenogenesis to osteogenesis [34]. Fibronectin and vitronectin have also been confirmed to induce chemotaxis and mitogenic activity of human and rabbit BMSCs [49]. In line with our findings, other group has demonstrated that these four ECM proteins are also preserved in the BMSC-derived ECM [16]. In the present study, only eight representative ECM components were selected to detect. There should be many other yet-to-be-detected bioactive components in the tECM-DTSs, which also may participate in regulating stem cell behavior.

BMSCs, as the most intensively used stem cells in tissue repair [48], have been proved to contribute to regeneration of various tissues, including tendon tissue [1, 50, 51]. Therefore, in the current study, BMSCs were selected as a test population to investigate the regulatory capacity of the tECM-DTSs to stem cell migration, proliferation and tenogenic differentiation. Encouragingly, the tECM-DTSs significantly promoted the migration of BMSCs. Our findings are in accordance with Lin’s report that the coating of urea-extracted fraction of human BMSC-derived ECM dramatically enhanced BMSCs migration in comparison to the coating of Type I collagen [52]. In addition, our recent work demonstrated that both BMSC-derived ECM-modified DTSs (bECM-DTSs) and tECM-DTSs obviously improved BMSCs migration by comparison with the DTSs; and the tECM-DTSs were significantly superior to the bECM-DTSs, which was probably caused by the release of significantly higher levels of chemokines in the extracts from the tECM-DTSs [27]. Unfortunately, only two chemokines, SDF-1 and monocyte chemotactic protein 1, were verified in these ECM-modified DTSs. In fact, except for these chemokines, multiple growth factors, just as TGF-β1, VEGF and IGF-1 [38–41, 45, 53], as well as some ECM proteins, like fibronectin and vitronectin [49], have also been confirmed to play considerable roles in promoting stem cell migration and recruitment. In addition to promoting the migration of BMSCs, also encouraging is that the tECM-DTSs significantly promoted the proliferation of BMSCs. Previous studies also reported that ECM deposited by SDSCs could serve for cell expansion system, which has dual function of improving the proliferation of the seeded cells and enhancing the chondrogenic potential of the expanded cells [13, 15, 54, 55]. DPSC-derived ECM for dental pulp regeneration has been shown to promote the proliferation of DPSCs *in vitro* [25]. In the current study, the alamarBlue assay revealed that the conditioned medium from the tECM-DTSs significantly promoted the proliferation of BMSCs in comparison to that from the DTSs. Although the soluble factors that released into the conditioned medium were not detected in this study, we believed the tECM-DTSs can release higher levels of cytokines than the DTSs, which play a critical role in facilitating BMSC proliferation. Due to the DTSs themselves with excellent ability in promoting stem cell proliferation [30], the seeded BMSCs grew robustly on the DTSs and tECM-DTSs, as well as maintained highly cell viability from 1 to 3 days, as indicated by the results of live/dead staining. Interestingly, the SEM images also showed that the tECM-DTSs remarkably promoted BMSCs proliferation. In the time-frame of 3 days, BMSCs on the tECM-DTSs rather than on the DTSs proliferated faster and completely covered the surface of scaffold material. Most strikingly, BMSCs could sense surface topographic differences between the DTSs and tECM-DTSs, and displayed random orientation on the tECM-DTSs without the highly aligned surface topographical cues. Moreover, as an ideal highly bioactive scaffold material for *in situ* tendon regeneration, recruiting abundant endogenous stem cells into the injury site and providing suitable microenvironment to promote cell proliferation are still not enough; further inducing the tenogenic differentiation of these recruited stem cells is also essential, which plays a critical role in tendon regeneration. Therefore, the scaffold with a greater capacity to induce stem cells tenogenic differentiation is highly desirable. In our previous study, we verified that the DTSs by a scaffold itself enhanced the tenogenic differentiation of rat TDSCs and BMSCs [30]. Promisingly, in the current study, the tECM-DTSs showed a greater capacity to induce BMSCs toward tenogenic differentiation compared to the DTSs, as evidenced by the results of RT-qPCR and western blot analysis. This finding strongly supports the view that ECM derived from stem cells maintain the functional properties of their native microenvironment and exhibit unique signaling that regulates stem cell self-renewal and lineage differentiation [14]. Indeed, in addition to serving as cell expansion system, stem cell-derived ECM can also act as cell differentiation inducers [14]. A previous study reported that differentiated BMSCs exhibited a rapid regression of osteoblastic markers upon the osteogenic cocktail removal but BMSC-derived ECM promoted the osteogenic potential of differentiated BMSCs in the absence of soluble osteoinductive cues, indicating the superiority of stem cell-derived ECM in inducing stem cell differentiation [22]. Though the intrinsic mechanisms are not fully understood, it is currently well accepted that ECM microenvironment cues, including but not limited to biochemical, topographical and biomechanical cues, play crucial roles in modulating stem cell fate. Interestingly, the tECM-DTSs with the modification of tECM on the DTSs substrate were found to change the highly aligned surface topographical cues of the DTSs and display an intricate and fibrillar ultrastructure. Although the topographical cues of scaffold materials that mimicking the aligned architecture of collagen fibers in tendons have been demonstrated to induce tenogenic differentiation of human TDSCs and human MSCs [56, 57], we cannot assert the surface topographical change caused by the modification of tECM will compromise the tenogenic differentiation of stem cells. Several studies have unveiled that induction of stem cells into a specific cell shape and arrangement is not consequentially accompanied by a lineage-specific differentiation [56, 58]. In the future, the role of the fibrillar ultrastructure of tECM in stem cell fate decision remains a subject for further investigation. As expected, the modification of tECM still retained the surface stiffness of the DTSs, which was about 1.2 MPa. After all, the stiffness of cell-derived ECM including MSC-derived ECM was only ∼0.1–1 kPa, as reported by Prewitz *et al*. [16]. Thus, the tECM-DTSs also had similar stiffness to native tendon, which may contribute to the tenogenic differentiation of BMSCs. Besides, most encouragingly, the modification of tECM significantly enhanced the content of multiple ECM components, including the two critical components (i.e. biglycan and fibromodulin) that controlled the tenogenic differentiation fate of TDSCs, which conferred higher bioactivity to the DTSs so that the tECM-DTSs had a greater capacity in inducing the tenogenic differentiation of BMSCs. In sum, these observations reveal the tremendous superiority of the scaffold materials consisting of tendon-specific tissue-derived ECM and stem cell-derived ECM in inducing the migration, proliferation as well as tenogenic differentiation of stem cells, which are hardly reproduced using single ECM proteins or synthetic scaffolds.

There are a few limitations to this study. First, a restricted number of biochemical components in the tECM-DTSs were investigated. Ongoing work will address this issue through comprehensive characterization of the critical bioactive components in the tECM-DTSs using proteomics analysis based on mass spectrometry. Second, the exact mechanism of the tECM-DTSs enhancing stem cell migration, proliferation and differentiation is not well understood. Further studies will focus on determining which of these ECM components are crucial for regulating stem cell behavior and analyzing the key signaling pathways to decipher how ECM components regulate stem cell function. Third, since the tECM-DTSs revealed a greater capacity to enhance the migration, proliferation as well as tenogenic differentiation of rat BMSCs compared to the DTSs, further studies are needed to investigate whether the tECM-DTSs are capable of recruiting abundant endogenous stem cells and inducing them toward tenogenic differentiation to promote *in situ* tendon regeneration.

## Conclusions

In summary, we developed a highly bioactive tendon-regenerative scaffold (i.e. tECM-DTSs) by surface modification of tissue-specific stem cell-derived ECM on the DTSs. The tECM-DTSs were found to change the highly aligned surface topographical cues of the DTSs, retain the stiffness of the DTSs and significantly increase the content of multiple ECM components. As a result, the tECM-DTSs dramatically enhanced the migration, proliferation as well as tenogenic differentiation of rat BMSCs compared with the DTSs. These findings further support the utilization of tissue-specific stem cell-derived ECM as a promising strategy to recapitulate the instructive stem cell microenvironment to enhance the bioactivity of scaffold materials.

## Supplementary data

Supplementary data are available at *REGBIO* online.

## Funding

This work was supported by the grants from National Natural Science Foundation of China (grant numbers: 32171349, 31600783 and 31870968), Science and Technology Plan of Sichuan Province (grant number: 2018SZ0044).

*Conflict of interest statement*. The authors have no conflicts of interest to declare.

## Supplementary Material

rbac020_Supplementary_Data

## References

[1] Ko IK , Lee SJ, Atala A, Yoo JJ. In situ tissue regeneration through host stem cell recruitment. Exp Mol Med 2013;45:e57. 24232256 10.1038/emm.2013.118 PMC3849571

[2] Gaharwar AK , Singh I, Khademhosseini A. Engineered biomaterials for in situ tissue regeneration. Nat Rev Mater 2020;5:686–705.

[3] Nover AB , Stefani RM, Lee SL, Ateshian GA, Stoker AM, Cook JL, Hung CT. Long-term storage and preservation of tissue engineered articular cartilage. J Orthop Res 2016;34:141–8. 26296185 10.1002/jor.23034 PMC4710567

[4] Xia H , Li X, Gao W, Fu X, Fang RH, Zhang L, Zhang K. Tissue repair and regeneration with endogenous stem cells. Nat Rev Mater 2018;3:174–93.

[5] Chen FM , Wu LA, Zhang M, Zhang R, Sun HH. Homing of endogenous stem/progenitor cells for in situ tissue regeneration: promises, strategies, and translational perspectives. Biomaterials 2011;32:3189–209. 21300401 10.1016/j.biomaterials.2010.12.032

[6] Thevenot PT , Nair AM, Shen J, Lotfi P, Ko CY, Tang L. The effect of incorporation of SDF-1alpha into PLGA scaffolds on stem cell recruitment and the inflammatory response. Biomaterials 2010;31:3997–4008. 20185171 10.1016/j.biomaterials.2010.01.144 PMC2838969

[7] Shi J , Sun J, Zhang W, Liang H, Shi Q, Li X, Chen Y, Zhuang Y, Dai J. Demineralized bone matrix scaffolds modified by CBD-SDF-1alpha promote bone regeneration via recruiting endogenous stem cells. ACS Appl Mater Interfaces 2016;8:27511–22. 27686136 10.1021/acsami.6b08685

[8] Kim JH , Jung Y, Kim BS, Kim SH. Stem cell recruitment and angiogenesis of neuropeptide substance P coupled with self-assembling peptide nanofiber in a mouse hind limb ischemia model. Biomaterials 2013;34:1657–68. 23206876 10.1016/j.biomaterials.2012.11.008

[9] Nair A , Shen J, Lotfi P, Ko CY, Zhang CC, Tang L. Biomaterial implants mediate autologous stem cell recruitment in mice. Acta Biomater 2011;7:3887–95. 21784181 10.1016/j.actbio.2011.06.050 PMC3185118

[10] Lu J , Shen X, Sun X, Yin H, Yang S, Lu C, Wang Y, Liu Y, Huang Y, Yang Z, Dong X, Wang C, Guo Q, Zhao L, Sun X, Lu S, Mikos AG, Peng J, Wang X. Increased recruitment of endogenous stem cells and chondrogenic differentiation by a composite scaffold containing bone marrow homing peptide for cartilage regeneration. Theranostics 2018;8:5039–58. 30429885 10.7150/thno.26981 PMC6217070

[11] Fitzpatrick LE , McDevitt TC. Cell-derived matrices for tissue engineering and regenerative medicine applications. Biomater Sci 2015;3:12–24. 25530850 10.1039/C4BM00246F PMC4270054

[12] Zhang W , Zhu Y, Li J, Guo Q, Peng J, Liu S, Yang J, Wang Y. Cell-derived extracellular matrix: basic characteristics and current applications in orthopedic tissue engineering. Tissue Eng Part B Rev 2016;22:193–207. 26671674 10.1089/ten.TEB.2015.0290

[13] Pei M , He F, Wei L. Three-dimensional cell expansion substrate for cartilage tissue engineering and regeneration: a comparison in decellularized matrix deposited by synovium-derived stem cells and chondrocytes. J Tissue Sci Eng 2011;2:104.

[14] Sart S , Jeske R, Chen X, Ma T, Li Y. Engineering stem cell-derived extracellular matrices: decellularization, characterization, and biological function. Tissue Eng Part B Rev 2020;26:402–22. 32220216 10.1089/ten.TEB.2019.0349

[15] Pei M , He F. Extracellular matrix deposited by synovium-derived stem cells delays replicative senescent chondrocyte dedifferentiation and enhances redifferentiation. J Cell Physiol 2012;227:2163–74. 21792932 10.1002/jcp.22950 PMC3265606

[16] Prewitz MC , Seib FP, von Bonin M, Friedrichs J, Stißel A, Niehage C, Müller K, Anastassiadis K, Waskow C, Hoflack B, Bornhäuser M, Werner C. Tightly anchored tissue-mimetic matrices as instructive stem cell microenvironments. Nat Methods 2013;10:788–94. 23793238 10.1038/nmeth.2523

[17] Assuncao M , Dehghan-Baniani D, Yiu CHK, Spater T, Beyer S, Blocki A. Cell-derived extracellular matrix for tissue engineering and regenerative medicine. Front Bioeng Biotechnol 2020;8:602009. 33344434 10.3389/fbioe.2020.602009 PMC7744374

[18] Gu Y , Li Z, Huang J, Wang H, Gu X, Gu J. Application of marrow mesenchymal stem cell-derived extracellular matrix in peripheral nerve tissue engineering. J Tissue Eng Regen Med 2017;11:2250–60. 26777754 10.1002/term.2123

[19] Yang Y , Lin H, Shen H, Wang B, Lei G, Tuan RS. Mesenchymal stem cell-derived extracellular matrix enhances chondrogenic phenotype of and cartilage formation by encapsulated chondrocytes in vitro and in vivo. Acta Biomater 2018;69:71–82. 29317369 10.1016/j.actbio.2017.12.043 PMC5831499

[20] Yan Y , Martin LM, Bosco DB, Bundy JL, Nowakowski RS, Sang QX, Li Y. Differential effects of acellular embryonic matrices on pluripotent stem cell expansion and neural differentiation. Biomaterials 2015;73:231–42. 26410789 10.1016/j.biomaterials.2015.09.020

[21] Sart S , Yan Y, Li Y, Lochner E, Zeng C, Ma T, Li Y. Crosslinking of extracellular matrix scaffolds derived from pluripotent stem cell aggregates modulates neural differentiation. Acta Biomater 2016;30:222–32. 26577988 10.1016/j.actbio.2015.11.016

[22] Hoch AI , Mittal V, Mitra D, Vollmer N, Zikry CA, Leach JK. Cell-secreted matrices perpetuate the bone-forming phenotype of differentiated mesenchymal stem cells. Biomaterials 2016;74:178–87. 26457835 10.1016/j.biomaterials.2015.10.003 PMC4661076

[23] Perez-Castrillo S , Gonzalez-Fernandez ML, Lopez-Gonzalez ME, Villar-Suarez V. Effect of ascorbic and chondrogenic derived decellularized extracellular matrix from mesenchymal stem cells on their proliferation, viability and differentiation. Ann Anat 2018;220:60–9. 30114449 10.1016/j.aanat.2018.07.006

[24] Nyambat B , Manga YB, Chen CH, Gankhuyag U, Pratomo WA, Kumar Satapathy M, Chuang EY. New insight into natural extracellular matrix: genipin cross-linked adipose-derived stem cell extracellular matrix gel for tissue engineering. Int J Mol Sci 2020;21:4864. 10.3390/ijms21144864 PMC7402347 32660134

[25] Zhang X , Li H, Sun J, Luo X, Yang H, Xie L, Yang B, Guo W, Tian W. Cell-derived micro-environment helps dental pulp stem cells promote dental pulp regeneration. Cell Prolif 2017;50:e12361. 10.1111/cpr.12361 PMC6529091 28741725

[26] Zhang W , Yang J, Zhu Y, Sun X, Guo W, Liu X, Jing X, Guo G, Guo Q, Peng J, Zhu X. Extracellular matrix derived by human umbilical cord-deposited mesenchymal stem cells accelerates chondrocyte proliferation and differentiation potential in vitro. Cell Tissue Bank 2019;20:351–65. 31218457 10.1007/s10561-019-09774-7

[27] Yao X , Ning LJ, He SK, Cui J, Hu RN, Zhang Y, Zhang YJ, Luo JC, Ding W, Qin TW. Stem cell extracellular matrix-modified decellularized tendon slices facilitate the migration of bone marrow mesenchymal stem cells. ACS Biomater Sci Eng 2019;5:4485–95. 33438414 10.1021/acsbiomaterials.9b00064

[28] He SK , Ning LJ, Yao X, Hu RN, Cui J, Zhang Y, Ding W, Luo JC, Qin TW. Hierarchically demineralized cortical bone combined with stem cell-derived extracellular matrix for regeneration of the tendon-bone interface. Am J Sports Med 2021;49:1323–32. 33667131 10.1177/0363546521994511

[29] Ning LJ , Zhang Y, Chen XH, Luo JC, Li XQ, Yang ZM, Qin TW. Preparation and characterization of decellularized tendon slices for tendon tissue engineering. J Biomed Mater Res A 2012;100:1448–56. 22378703 10.1002/jbm.a.34083

[30] Ning LJ , Zhang YJ, Zhang Y, Qing Q, Jiang YL, Yang JL, Luo JC, Qin TW. The utilization of decellularized tendon slices to provide an inductive microenvironment for the proliferation and tenogenic differentiation of stem cells. Biomaterials 2015;52:539–50. 25818459 10.1016/j.biomaterials.2015.02.061

[31] Pan J , Liu GM, Ning LJ, Zhang Y, Luo JC, Huang FG, Qin TW. Rotator cuff repair using a decellularized tendon slices graft: an in vivo study in a rabbit model. Knee Surg Sports Traumatol Arthrosc 2015;23:1524–35. 24623185 10.1007/s00167-014-2923-7

[32] Ning LJ , Jiang YL, Zhang CH, Zhang Y, Yang JL, Cui J, Zhang YJ, Yao X, Luo JC, Qin TW. Fabrication and characterization of a decellularized bovine tendon sheet for tendon reconstruction. J Biomed Mater Res A 2017;105:2299–311. 28380688 10.1002/jbm.a.36083

[33] Yang B , Zhang Y, Zhou L, Sun Z, Zheng J, Chen Y, Dai Y. Development of a porcine bladder acellular matrix with well-preserved extracellular bioactive factors for tissue engineering. Tissue Eng Part C Methods 2010;16:1201–11. 20170425 10.1089/ten.TEC.2009.0311

[34] Bi Y , Ehirchiou D, Kilts TM, Inkson CA, Embree MC, Sonoyama W, Li L, Leet AI, Seo BM, Zhang L, Shi S, Young MF. Identification of tendon stem/progenitor cells and the role of the extracellular matrix in their niche. Nat Med 2007;13:1219–27. 17828274 10.1038/nm1630

[35] Tan Q , Lui PP, Rui YF, Wong YM. Comparison of potentials of stem cells isolated from tendon and bone marrow for musculoskeletal tissue engineering. Tissue Eng Part A 2012;18:840–51. 22011320 10.1089/ten.tea.2011.0362 PMC3313612

[36] Crapo PM , Gilbert TW, Badylak SF. An overview of tissue and whole organ decellularization processes. Biomaterials 2011;32:3233–43. 21296410 10.1016/j.biomaterials.2011.01.057 PMC3084613

[37] Pei M , He F, Kish VL. Expansion on extracellular matrix deposited by human bone marrow stromal cells facilitates stem cell proliferation and tissue-specific lineage potential. Tissue Eng Part A 2011;17:3067–76. 21740327 10.1089/ten.tea.2011.0158 PMC3226057

[38] Zhang F , Tsai S, Kato K, Yamanouchi D, Wang C, Rafii S, Liu B, Kent KC. Transforming growth factor-beta promotes recruitment of bone marrow cells and bone marrow-derived mesenchymal stem cells through stimulation of MCP-1 production in vascular smooth muscle cells. J Biol Chem 2009;284:17564–74. 19406748 10.1074/jbc.M109.013987 PMC2719395

[39] Gao P , Zhou Y, Xian L, Li C, Xu T, Plunkett B, Huang SK, Wan M, Cao X. Functional effects of TGF-beta1 on mesenchymal stem cell mobilization in cockroach allergen-induced asthma. J Immunol 2014;192:4560–70. 24711618 10.4049/jimmunol.1303461 PMC4039654

[40] Si X , Liu X, Li J, Wu X. Transforming growth factor-beta1 promotes homing of bone marrow mesenchymal stem cells in renal ischemia-reperfusion injury. Int J Clin Exp Pathol 2015;8:12368–78. 26722423 PMC4680368

[41] Zhang SJ , Song XY, He M, Yu SB. Effect of TGF-beta1/SDF-1/CXCR4 signal on BM-MSCs homing in rat heart of ischemia/perfusion injury. Eur Rev Med Pharmacol Sci 2016;20:899–905. 27010148

[42] Dubon MJ , Yu J, Choi S, Park KS. Transforming growth factor beta induces bone marrow mesenchymal stem cell migration via noncanonical signals and N-cadherin. J Cell Physiol 2018;233:201–13. 28213973 10.1002/jcp.25863

[43] Zhang F , Ren T, Wu J, Niu J. Small concentrations of TGF-beta1 promote proliferation of bone marrow-derived mesenchymal stem cells via activation of Wnt/beta-catenin pathway. Indian J Exp Biol 2015;53:508–13. 26349313

[44] Sun J , Zhou Y, Ye Z, Tan WS. Transforming growth factor-beta1 stimulates mesenchymal stem cell proliferation by altering cell cycle through FAK-Akt-mTOR pathway. Connect Tissue Res 2019;60:406–17. 30642198 10.1080/03008207.2019.1570171

[45] Ball SG , Shuttleworth CA, Kielty CM. Vascular endothelial growth factor can signal through platelet-derived growth factor receptors. J Cell Biol 2007;177:489–500. 17470632 10.1083/jcb.200608093 PMC2064818

[46] Haider H , Jiang S, Idris NM, Ashraf M. IGF-1-overexpressing mesenchymal stem cells accelerate bone marrow stem cell mobilization via paracrine activation of SDF-1alpha/CXCR4 signaling to promote myocardial repair. Circ Res 2008;103:1300–8. 18948617 10.1161/CIRCRESAHA.108.186742

[47] Shen W , Chen X, Chen J, Yin Z, Heng BC, Chen W, Ouyang HW. The effect of incorporation of exogenous stromal cell-derived factor-1 alpha within a knitted silk-collagen sponge scaffold on tendon regeneration. Biomaterials 2010;31:7239–49. 20615544 10.1016/j.biomaterials.2010.05.040

[48] Fu X , Liu G, Halim A, Ju Y, Luo Q, Song AG. Mesenchymal stem cell migration and tissue repair. Cells 2019;8:784. 10.3390/cells8080784 PMC6721499 31357692

[49] Thibault MM , Hoemann CD, Buschmann MD. Fibronectin, vitronectin, and collagen I induce chemotaxis and haptotaxis of human and rabbit mesenchymal stem cells in a standardized transmembrane assay. Stem Cells Dev 2007;16:489–502. 17610379 10.1089/scd.2006.0100

[50] Cai J , Yang Y, Ai C, Jin W, Sheng D, Chen J, Chen S. Bone marrow stem cells-seeded polyethylene terephthalate scaffold in repair and regeneration of rabbit achilles tendon. Artif Organs 2018;42:1086–94. 30294929 10.1111/aor.13298

[51] Xie S , Zhou Y, Tang Y, Chen C, Li S, Zhao C, Hu J, Lu H. Book-shaped decellularized tendon matrix scaffold combined with bone marrow mesenchymal stem cells-sheets for repair of achilles tendon defect in rabbit. J Orthop Res 2019;37:887–97. 30816590 10.1002/jor.24255

[52] Lin H , Yang G, Tan J, Tuan RS. Influence of decellularized matrix derived from human mesenchymal stem cells on their proliferation, migration and multi-lineage differentiation potential. Biomaterials 2012;33:4480–9. 22459197 10.1016/j.biomaterials.2012.03.012

[53] Xinaris C , Morigi M, Benedetti V, Imberti B, Fabricio AS, Squarcina E, Benigni A, Gagliardini E, Remuzzi G. A novel strategy to enhance mesenchymal stem cell migration capacity and promote tissue repair in an injury specific fashion. Cell Transplant 2013;22:423–36. 22889699 10.3727/096368912X653246

[54] He F , Chen X, Pei M. Reconstruction of an in vitro tissue-specific microenvironment to rejuvenate synovium-derived stem cells for cartilage tissue engineering. Tissue Eng Part A 2009;15:3809–21. 19545204 10.1089/ten.TEA.2009.0188

[55] Li J , Pei M. Optimization of an in vitro three-dimensional microenvironment to reprogram synovium-derived stem cells for cartilage tissue engineering. Tissue Eng Part A 2011;17:703–12. 20929284 10.1089/ten.TEA.2010.0339

[56] Yin Z , Chen X, Chen JL, Shen WL, Hieu Nguyen TM, Gao L, Ouyang HW. The regulation of tendon stem cell differentiation by the alignment of nanofibers. Biomaterials 2010;31:2163–75. 19995669 10.1016/j.biomaterials.2009.11.083

[57] Kishore V , Bullock W, Sun X, Van Dyke WS, Akkus O. Tenogenic differentiation of human MSCs induced by the topography of electrochemically aligned collagen threads. Biomaterials 2012;33:2137–44. 22177622 10.1016/j.biomaterials.2011.11.066 PMC3279298

[58] Tong WY , Shen W, Yeung CW, Zhao Y, Cheng SH, Chu PK, Chan D, Chan GC, Cheung KM, Yeung KW, Lam YW. Functional replication of the tendon tissue microenvironment by a bioimprinted substrate and the support of tenocytic differentiation of mesenchymal stem cells. Biomaterials 2012;33:7686–98. 22818988 10.1016/j.biomaterials.2012.07.002

