# Supplementary material for: Constructing a highly bioactive tendon-regenerative scaffold by surface modification of tissue-specific stem cell-derived extracellular matrix
Source: Regen Biomater. 2022 Apr 20;9:rbac020. doi: 10.1093/rb/rbac020 (PMC9036902; doi:10.1093/rb/rbac020)
Supplement: rbac020_Supplementary_Data [file rbac020_supplementary_data.doc]

**Supplemental Figure**


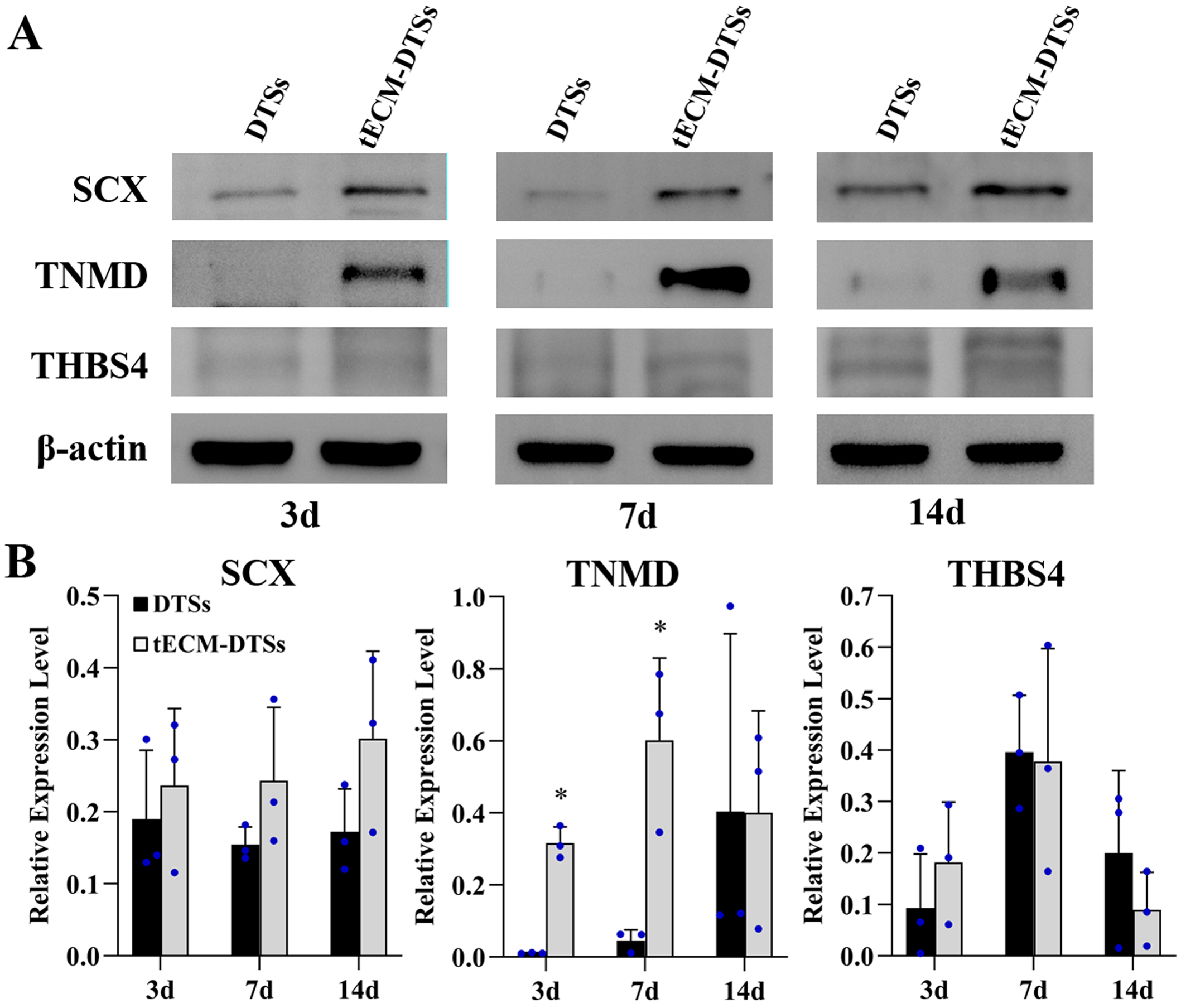


Figure S1. Cell differentiation assays of the DTSs and tECM-DTSs on the protein expression level. (A) Representative western blot bands of BMSCs cultured on the DTSs and tECM-DTSs at 3, 7, and 14 d. (B) Semi-quantitative analysis of tendon-specific proteins expression of BMSCs cultured on the DTSs and tECM-DTSs at different time points. Data are normalized to β-actin *, *p* <0.05 as compared with the DTSs.
